# Tocilizumab and COVID-19: Timing of Administration and Efficacy

**DOI:** 10.3389/fphar.2022.825749

**Published:** 2022-02-18

**Authors:** Emna Abidi, Wasim S. El Nekidy, Eman Alefishat, Nadeem Rahman, Georg A. Petroianu, Rania El-Lababidi, Jihad Mallat

**Affiliations:** ^1^ Department of Pharmacy Services, Cleveland Clinic Abu Dhabi, Abu Dhabi, United Arab Emirates; ^2^ Cleveland Clinic Lerner College of Medicine of Case Western Reserve University, Cleveland, OH, United States; ^3^ Department of Pharmacology, College of Medicine and Health Science, Khalifa University, Abu Dhabi, United Arab Emirates; ^4^ Department of Biopharmaceutics and Clinical Pharmacy, Faculty of Pharmacy, The University of Jordan, Amman, Jordan; ^5^ Center for Biotechnology, Khalifa University of Science and Technology, Abu Dhabi, United Arab Emirates; ^6^ Critical Care Institute, Cleveland Clinic Abu Dhabi, Abu Dhabi, United Arab Emirates; ^7^ Normandy University, UNICAEN, Caen, France

**Keywords:** COVID-19 infection, SARS-CoV-2 infection, cytokine storm, interleukine -6 receptor antagonist, tocilizumab, acute respiratory distress syndrome, invasive mechanical ventilation, intensive care unit

## Abstract

Elevated concentrations of interleukin-6 have been demonstrated to be an important key factor in COVID-19 host immune impairment. It represents an important prognostic factor of harm associated with COVID-19 infection by stimulating a vigorous proinflammatory response, leading to the so-called “cytokine storm”. Therefore, immunomodulatory interventions targeting interleukin-6 receptor antagonism have been investigated as potential treatments to counterbalance the host immune dysregulation and to support the advantageous effects of corticosteroids. Tocilizumab is a recombinant humanized monoclonal antibody that has gained much interest during the COVID-19 pandemic as an interleukin-6 receptor antagonist. Various early observational studies have reported beneficial effects of tocilizumab. Moreover, consequent randomized controlled trials have subsequently shown significant positive results about tocilizumab efficacy and safety, focusing on outcomes like mortality, risk of intensive care unit admission, and the need for mechanical ventilation, while others presented conflicting findings. In this review, we first described the pathophysiology of COVID-19 infection while highlighting the role of interleukin-6. Furthermore, we also discussed the non-conclusive evidence about tocilizumab to be used as the standard of care therapy for all patients with COVID-19 pneumonia, as well as its beneficial effects in selected patients.

## 1 Introduction

COVID-19 was recognized as a pandemic by the World Health Organization (WHO) in March 2020 (World Health Organization, 2020). Since it first started in Wuhan, COVID-19 has been confirmed in over two hundred twenty-three million cases and resulted in over four million deaths globally as of September 2021 (WHO). The pandemic has been inflicting unprecedented harm on the economic and health sectors (Zhou et al., 2020a). Therefore, intensive worldwide efforts have been largely demonstrated towards the development of effective therapy that can reduce risk of severe COVID-19 diseases, hospitalizations, and death.

Massive ground-breaking research efforts resulted in the development of a variety of about 12 recently available and approved vaccines to prevent the uncontrollable worldwide spread of SARS-CoV-2 virus (Marian, 2021). In addition to vaccine development, parallel efforts are still ongoing to develop effective therapy in order to reduce the risk of progression to severe disease and reduce mortality (Walls et al., 2020; Yu et al., 2020). Different therapeutic strategies have been employed to treat patients with COVID-19 and prevent the progression of the disease (Izda et al., 2021). Most recently, immunomodulation has been shown to be a highly effective as largely demonstrated by corticosteroids (Sterne et al., 2020). Tocilizumab is a highly specific monoclonal antibody that works as an immunomodulator directly targeting interleukin-6 (IL-6) via the IL-6 receptor (IL-6R). Although the effectiveness of tocilizumab on the SARS-CoV-2-triggered immune response has been proven, its usage as a sole drug and the optimal timing to introduce it to the treatment regimen remains controversial (Huang and Jordan, 2020; Tsai et al., 2020). In this review, the pathophysiology of COVID-19-associated cytokine release syndrome, the pharmacology of tocilizumab and its impact on the cytokine release syndrome, clinical studies investigating its use in COVID-19 with respect to timing of administration in the disease continuum are discussed.

## 2 COVID-19 and Cytokine Release Syndrome

### 2.1 COVID-19 Mechanisms of Infection

**FIGURE 1:**
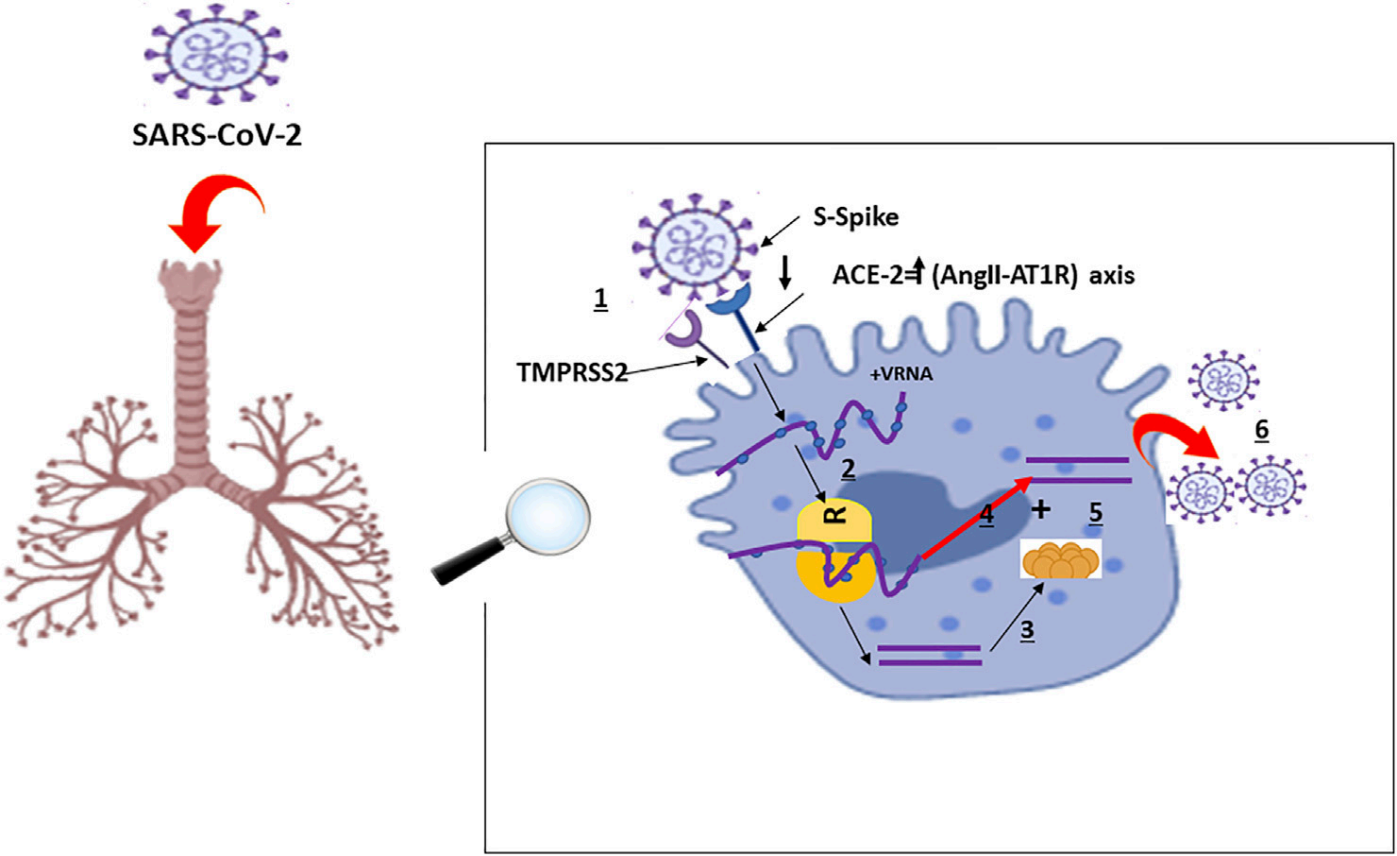
Schematic Representation of Physiological Host Response to SARS-CoV-2 Infection with A and B representing the disease severity and related events: A: Viral entry and early infection (Proptosis induction): (World Health Organization, 2020): SARS-CoV-2 viral infection of alveolar epithelial cells via surface binding of spike (S) protein to angiotensin converting enzyme 2 facilitated by transmembrane serine protease 2: **SARS-CoV-2:** Severe Acute Respiratory Syndrome Coronavirus 2; **TMPRSS2:** S protein priming; **ACE-2**: Angiotensin-Converting Enzyme 2; **AngII-AT1R:** AngII-angiotensin type 1 receptor; **VRNA**: Viral Ribonucleic Acid; **R**: Ribosome; **1:** Fusion**; 2:** Translation; **3**: Proteolysis; **4**: Translation and RNA replication; **5**: Packaging; **6**: Virion release.

**FIGURE 2:**
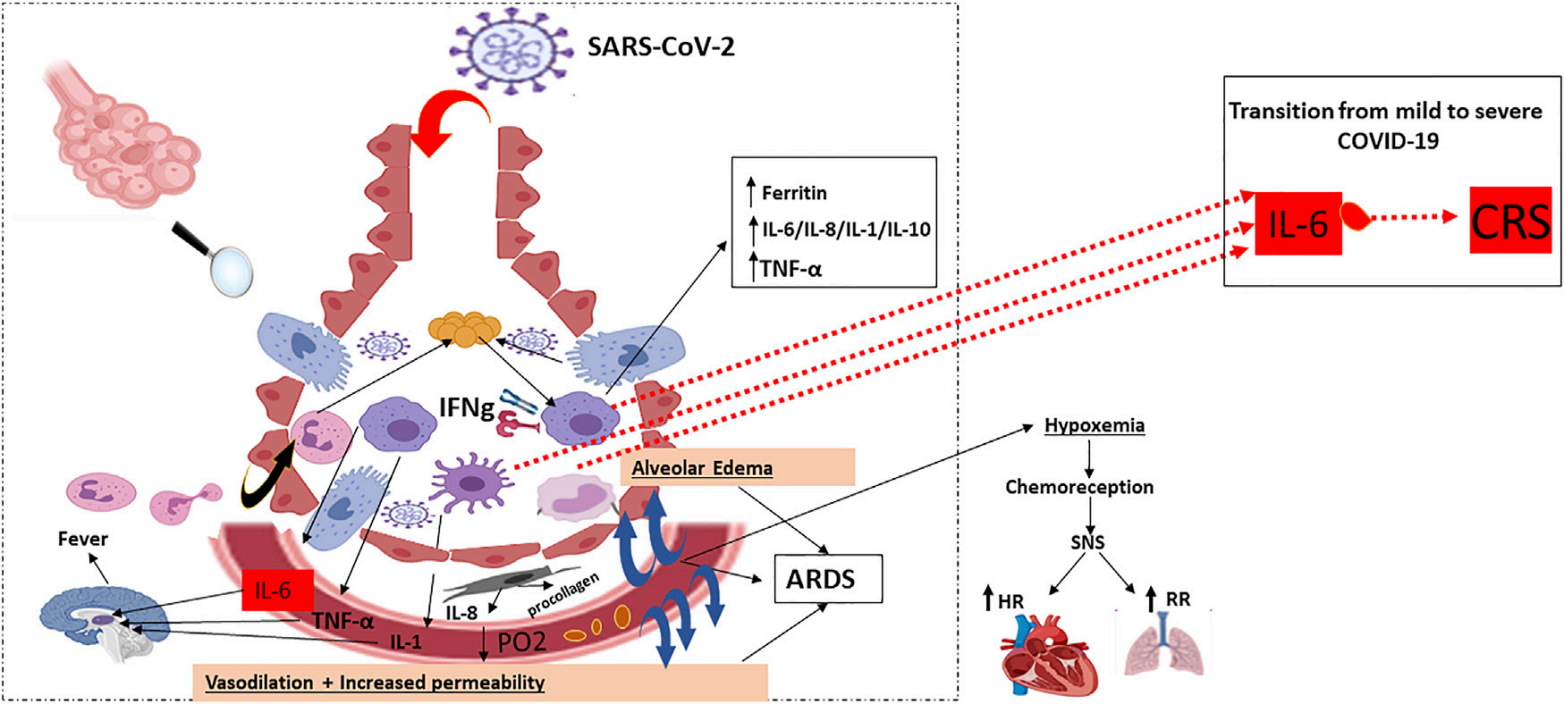
Host immune response: pulmonary recruitment of macrophages and dendritic cells in response to chemokine and cytokine release: Characterised by DAMP/PAMPs recognition, pro-inflammatory cytokine and chemokine release (early phase), activation of different cells of the mononuclear phagocyte system and virus specific cytotoxic T cells recruitment (late phase). Activated macrophages, monocytes and dendritic cells secrete IL-6 in increased quantities: **IFN-γ**: Interferon-γ; **TNF-α:** Tumor necrosis factor-α; **ARDS:** Acute Respiratory Distress Syndrome; **PO2**: Partial Pressure of Oxygen; **SNS**: Somatic Nervous System; **HR**: Heart Rate; **RR**: Respiratory Rate; **IL**: Interleukin; **CRS**: **Cytokine Release Syndrome.** 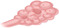: Alveoli Cross section; 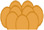: Viral proteins; 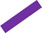: Polypeptide chain; 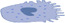: Respiratory Epithelial cell; 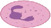: Activated Neutrophil; 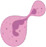: Migrating Neutrophil; 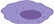: Activated Macrophage; 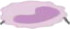: Activated Monocyte; 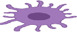: Activated Dendritic cell; 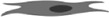: Fibroblast; 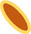: Platelets.

The RNA virus, SARS-CoV-2, binds via its “spike protein” to the angiotensin-converting enzyme 2 (ACE2) receptor expressed in cardiopulmonary tissues and in some hematopoietic cells, including monocytes and macrophages The serine protease TGRBSS2 has been reported to facilitate SARS-CoV-2 entry into the host cells (Figure 1) (Matricardi et al., 2020; Zhou et al., 2020b; Hoffmann et al., 2020). Once inside, SARS-CoV-2 incites an immune response mainly marked by orchestral responses taking place downstream the membrane-bound immune receptors (e.g., Fc and Toll-like receptors) and the downstream proinflammatory signaling pathways. A prompt activation of, both, pathogenic Th1 cells that release proinflammatory cytokines, is then mainly marked by a remarkable secretion of granulocyte-macrophage colony-stimulating factor (GM-CSF), interleukin-6 (IL-6) and intermediate inflammatory CD14^+^ CD16^+^monocytes. Further activation of those latter by GM-CSF produce much more amounts of IL-6, tumor necrosis factor-α (TNF-α) cytokines, among others (Haiming et al., 2020) (Figure 2). In the context of SARS-CoV-2 infection, the inflammatory cascade is activated in an imbalanced manner. This includes a reduced IFN-γ secretion hat results in amplified cytokine production (Hussman, 2020) enhanced on its turn by the extracellular grid called Neutrophil extracellular traps (NETs) (Zuo et al., 2020). The snow-ball effect of releasing much more cytokines, leads to the so-called “Cytokine storm,” mainly characterized by an extensive expression of IL-6 and TNF-α (Hirano and Murakami, 2020; Hussman, 2020).

The pathophysiology of the SARS-CoV-2 related cytokine storm, caused by the angiotensin 2 (AngII) pathway, has not been largely investigated. However, some authors tried to potentially describe it as follows: SARS-CoV-2 occupies the ACE2 receptors on the host cell surfaces via NF-κB recognition receptors (PPRs) activation. ACE2 saturation results, then, in a reduction of its proper expression which results in a subsequent rise in Ang II production necessary for AngII-angiotensin receptor type 1 (AngII-AT1R) axis activation. AngII-AT1R 1 axis can also induceTNF-α and soluble form of IL-6Ra (sIL-6Ra) via disintegrin and metalloprotease 17 (ADAM17) (Eguchi et al., 2018) (Figures 1, 2).

### 2.2 IL-6 the Orchestral Lead for COVID-19 Cytokine Release Syndrome

An important factor in the subsequent inflammatory reaction exacerbation is IL-6. It acts in a tran-signaling pathway manner via the, ubiquitously expressed gp130, to bind to the sIL-6R and form the IL-6-sIL-6R complex on almost all cell surfaces. IL-6 acts on its own production favor by many means to maintain the loop effect of pro-inflammatory exacerbation. Another signaling pathway for IL-6 is the *cis*-mediated signaling via the membrane-bound IL-6 receptor (mIL-6R) restrictedly expressed on immune cells. However, the pleiotropic effects of IL-6 pathways on the immune system, both the uncontrolled innate responses (neutrophils, macrophages, and natural killer (NK) cells), and the impaired inquired immune responses (B and T cells) have been attributed mainly to the *cis*-signaling pathway being the most responsible of the described SARS-CoV-2-related cytokine release syndrome (CRS) (Moore and June 2020a).

The resultant IL-6–sIL-6R–JAK-STAT3 association is primordial for activating the JAKs (Janus kinases) STAT3 (signal transducer and activator of transcription 3) pathway even in non-immune cells that do not express mIL-6R, such as the endothelial cells. Positive feedback is then enhanced by both STAT3 and NF-κB to multiply the IL-6 secretion via the IL-6 amplifier (IL-6 Amp) activation.

**FIGURE 3:**
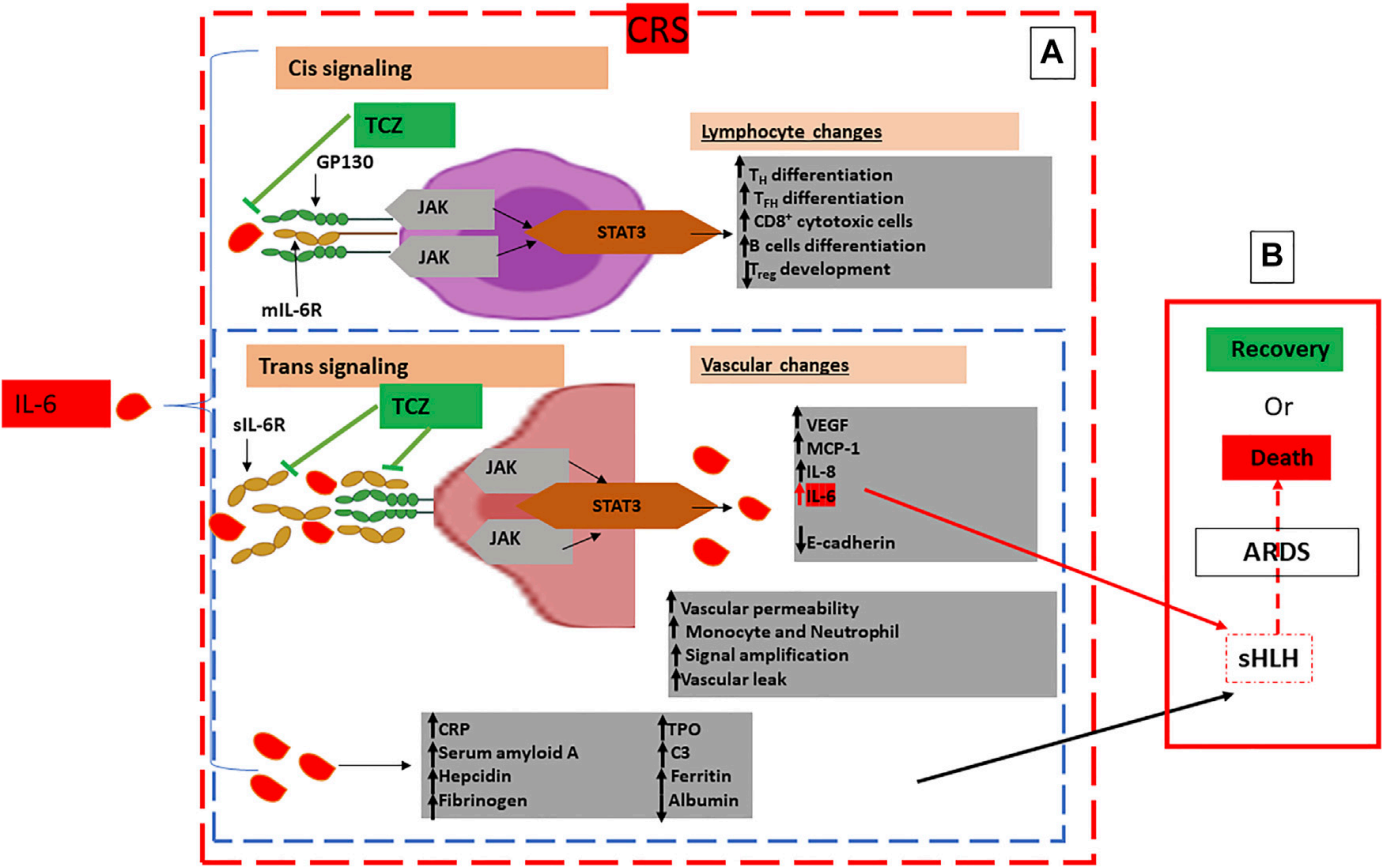
Schematic Representation of Pathogenic Host Response to SARS-CoV-2 and Pathways to Cytokine Release Syndrome (CRS): **(A)**: Hyperinflammatory phase: CRS characterised by excessive concentrations of IL-6 leading to an amplified IL-6–sIL-6R–JAK-STAT3 Cis and Trans signaling pathways with, respectively, lymphocyte and vascular changes in multiple cell types such as lymphocytes and endothelial cells characterised by excessive infiltration of immune cells in the lungs, systemic overproduction of pro-inflammatory cytokines and aberrant regulation. **(B)**: Multiorgan dysfunction: Characterised by extrapulmonary organs involvement, sHLH, ARDS and ultimately to COVID-19 patients’ death. Immunomodulatory treatment by IL-6 antagonists is represented by TCZ directed against both the soluble and the membrane-bound forms of IL-6R to inhibit both CIS and Trans singling pathways: **Glycoprotein 130: gp 130; mIL-6R**: membrane-bound IL-6 receptor; **sIL-6R**: soluble IL-6 receptor; **JAK**: Janus kinase; **MCP-1**: Monocyte Chemoattractant Protein–1; **STAT3**: Signal Transducer and Activator of Transcription 3; **T**_**FH**_: T follicular helper cell; **T**_**H17**_: T helper 17 cell; **TPO**: Thrombopoietin; **T**_**reg**_: T regulatory cell; **VEGF**: Vascular Endothelial Growth Factor; **C3**: Complement 3; **CRP**: C Reactive Protein; **TCZ**: Tocilizumab; 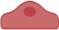: Endothelial cell; 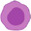: Lymphocyte.

Amplifying IL-6 production plays a crucial role to induce a panoply of proinflammatory cytokines and chemokines such as the endothelial growth factor (VEGF), monocyte chemoattractant protein-1 (MCP-1), IL-8, as well as reduced E-cadherin expression on endothelial cells and the IL-6, always produced in advantage, resulting in systemic “Cytokine storm” (Murakami et al., 2019) (Figure 3A). In this context, both VEGF and lowered E-cadherin expression cause vascular permeability and outflow playing, then a prominent role in the pathophysiology of hypotension and pulmonary dysfunction primarily observed in the acute respiratory distress syndrome (ARDS) (Figure 3B). Despite the rapidly developing pandemic, SARS-CoV-2 infection severity still conserves its main features that are fever and pneumonia, the foremost leaders to ultimate respiratory deterioration marked by ARDS observed in more than 20% of COVID-19 cases. This being said, CRS is common in patients with COVID-19, and most importantly, high serum IL-6 constitutes a hallmark of respiratory failure, ARDS, and the worst clinical outcomes.

## 3 Temporal Development of CRS and COVID-19 Clinical Gravity

SARS-CoV-2 viral infection can be asymptomatic and/or present with mild upper respiratory symptoms. However, cases of severe pneumonia and ARDS require care in the intensive care setting and a high likelihood of receiving oxygen support or mechanical ventilatrion.

This has been recently attributed to different balances between the viral load, on the one hand, and the efficacy of the local innate response (IgA, IgM, MBL antibodies) on the other hand, which is crucial to determining the prognosis of COVID-19 patients (Tsai et al., 2020).

Some studies found that patients with severe COVID-19 exhibit higher levels of IL-2, IL-7, IL-10, Interferon gamma-induced protein 10 (IP-10), macrophage inflammatory protein 1 alpha (MIP1A), and granulocyte-colony stimulating factor (G-CSF) than patients with mild and moderate infections (Tay et al., 2020). In fact, severe cases of COVID-19 characterized by CRS present often with systemic and local complications, such as venous vasculopathy in the lungs accompanied by multiple thrombotic small vessels, lung lesions, and ultimately lead to ARDS (Tsai et al., 2020). Other frequent complications have mainly been described at this stage, with cardiac and renal damage as the most reported (Huang et al., 2020).

Furthermore, severe COVID-19 have also been related to cases of cytopenia marked by an accentuated and continuous drop in lymphocytes counts; precisely much decreased CD8^+^ T cells count, leading to increased inflammatory response, compared to CD4^+^ and an increased neutrophil count when compared to mild patients (Liu et al., 2020). Thus, both T cell lymphocytopenia and active CRS are related to a severe COVID-19 leading to long-term damage and fibrosis of lung tissues (Figure 3A) (Pedersen and Ho, 2020).

The temporal evolution of both the dynamic cytokine reactions and lymphocytes count is a good clinical marker for clinicians to spot patients at higher risk of COVID-19 severe prognosis and to monitor both parameters for better management of COVID-19 cases severity (Ong et al., 2020). Some quantifiable markers such as D-Dimer and C-reactive protein are also used to predict a patient’s prognosis (Eparine a basso peso molecolare, 2020). Moreover, an association between elevated serum ferritin and IL-6, characteristic of the sHLH, and higher mortality rates have been reported in COVID-19 patients (Ruan et al., 2020).

Therefore, the sHLH that escorts serious COVID-19 with a well-defined CRS-induced ARDS urgently needs therapeutics interventions based on suppressing CRS, like glucocorticoids and some IL-6R blockers such as tocilizumab (Figure 3A).

## 4 Immunomodulatory Treatment of COVID-19

Pathological features and clinical stages of the COVID-19 have led various researchers towards different hypotheses to test potential treatments. Several drug classes have been tested in the management of COVID-19, including antiviral agents in the early stages, inflammation inhibitors, low-molecular-weight heparins, as well as different immunomodulators such as colchicine, inhaled Corticosteroids (Budesonide, Ciclesonide), Fluvoxamine, Il-1 inhibitors (Anakinra, Canakinumab), anti-Interleukin-6 monoclonal antibodies (Siltuximab), Non-Sars-CoV-2 specific immunoglobulin and Bruton’s tyrosine kinase (BTK) inhibitors, all of which are currently under investigation in clinical trials, not yet approved by the FDA and not recommended by the panel for COVID-19 treatments. Other classes of immunomodulators such as systemic corticosteroids (dexamethasone), some Kinase inhibitors like Janus Kinase inhibitors (JAK) (Baricitinib, Tofacitinib) are recommended by the panel for the treatment of COVID-19 in certain nonhospitalized and hospitalized patients (Characteristics of Immunomodulators, 2022). Moreover, convalescent plasma has been suggested and approved for the treatment of COVID-19 based on the experience gathered by treating influenza, Ebola and SARS (Luke et al., 2010; Chen et al., 2020a). Beside the above cited immunomodulators, IL-6 constitutes a target in multiple therapeutic strategies, such as IL-6R antagonists, that have already gained much focus for treating different scenarios of CRS and sHLH SARS (Channappanavar and Perlman, 2017), notably in COVID-19. Accordingly, in the context of SARS-CoV-2, much attention has been focused on demonstrating the efficacy of anti-IL-6 Monoclonal Antibodies (sarilumab and tocilizumab) immunomodulation by reversing IL-6 pathways, since drastically elevated concentrations of IL-6 in serum have been associated with higher levels of SARS-CoV-2 viremia (Chen et al., 2020b), longer viral RNA shedding (Lin et al., 2020), higher risk for mechanical ventilation (Herold et al., 2020) and mortality (Del Valle et al., 2020a).

Tocilizumab is a recombinant humanized monoclonal antibody of the IgG1 class, directed against both the soluble and the membrane-bound forms of IL-6R (Figure 3A). Both early and ongoing research initiatives have demonstrated that IL-6R blockade can interrupt the inflammatory cascade at a crucial stage (Guaraldi et al., 2020a; Gupta et al., 2021a). However, randomized control trials (RCTs) have generated contradictory reports (Hermine et al., 2021; Veiga et al., 2021). We report and discuss major RCTs regarding the necessity and efficacy of using tocilizumab as a drug of preference to treat COVID-19 patients. We focused on discussing the rationale behind using tocilizumab, as well as differences in its use based on the COVID-19 stage and, consequently, the patient’s status.

### 4.1 Tocilizumab Efficacy and Safety to Face the COVID-19 Cytokine Release Syndrome

As a well-known agent for IL-6 blockade, tocilizumab was enthusiastically used first in the United States, despite limited literature citing benefit in COVID-19 patients (FDA NEWS RELEASE, 2021). However, many observational studies, followed by RCTs, have recently assessed the safety and efficacy of using tocilizumab in COVID-19 management.

A small-sample clinical trial performed in China documented the effectiveness of tocilizumab in COVID-19 cases (Chen et al., 2020c), with a 90% recovery following a short period of the treatment in critically ill patients (Xu et al., 2020). Thereafter, some case-control retrospective studies have reported the possibility of tocilizumab to decrease mortality rates in most critical COVID-19 patients with developed ARDS (Wadud et al., 2020).

More retrospective case-control studies performed in COVID-19 patients with ARDS suggested the possibility of tocilizumab to improve the survival outcome (Zhang et al., 2020a; Cellina et al., 2020). In severe COVID-19 pneumonia patients, the use of tocilizumab was shown to be significantly associated with a reduced risk of invasive mechanical ventilation or death (Somers et al., 2021).

The safety and efficacy of tocilizumab have been assessed in larger and different cohorts of COVID-19 patients at different stages of the infection. The most worth citing RCTs that, mainly, have driven much more insights into the matter are the landmark RECOVERY (RECOVERY Collaborative GroupHorby et al., 2021), the Randomized, Embedded, Multifactorial Adaptive Platform Trial for Community-Acquired Pneumonia (REMAP-CAP) platform (Gordon et al., 2021), the COVACTA (Rosas et al., 2021) and the EMPACTA studies (Salama et al., 2021).

RCTs mentioned above have discussed their results across different groups of patients with differing disease severity. Therefore, conflicting results were potentially due to differences in timings of tocilizumab administartion having impacted the observed clinical outcomes.

Hence, the following discussion of tocilizumab efficacy will be considering when and to which patient the drug has been administered, with its efficacy evaluated based on clinical outcomes.

### 4.2 Timing of Administration of Tocilizumab in Moderately Ill COVID-19 Patients

#### 4.2.1 Early Administration to Prevent the Cytokine Release Syndrome

Stone et al. (Stone et al., 2020) conducted a randomized, double-blind, placebo-controlled trial on 243 moderately ill COVID-19 patients with hyperinflammatory state (C-reactive protein >50 mg/L, or ferritin >500 ng/ml, or D-dimer > 1,000 ng/ml, or lactate dehydrogenase >250 U/L), fever (body temperature >38°C), pulmonary infiltrates, or the need for supplemental oxygen (<10 L/min) in order to maintain an oxygen saturation >92%. The median time from symptom onset to tocilizumab treatment was 9 (Walls et al., 2020; Izda et al., 2021; Sterne et al., 2020; Tsai et al., 2020; Huang and Jordan, 2020; Matricardi et al., 2020; Zhou et al., 2020b; Hoffmann et al., 2020) days. The study hypothesis was based on the possibility of blocking IL-6R at the early stages of the disease before patients progress into the severe stages of the disease, thus halting the associated inflammatory status from progressing to CRS. However, tocilizumab treatment was not found to be effective in preventing intubation or death within 28 days, nor for stopping disease progression (Table 1). Thus, the authors failed to demonstrate the benefit of tocilizumab use as a treatment strategy to avoid the evolution of COVID-19 from a moderate to a severe state. Besides the absence of efficacy proofs, the described study demonstrated the absence of serious side effects in the study group while mild-moderate infections were seen in the tocilizumab treated group (Stone et al., 2020).

**TABLE 1:** A Selective List of Randomized Clinical Trials Highlighting the Efficacy, as well as Non-efficacy, of Tocilizumab in COVID-19 Patients at Different Stages of the Infection Severity.

NO tocilizumab efficacy: RCTs evidences							
COVID-19 infection status	Number/Ageof participants	Time/Dose/route of TCZ use	Concomitant treatments	Primary efficacy outcomes	Secondary and tertiary efficacy outcomes	Safety	SR
Moderate infection	Total patients 243	-Within 10 days after the COVID-19 symptoms onset. -Single IV: 8 mg/kg	AV, HCQ, and GCC.	MV/Death: 10.6% in the TCZ gp and 12.5% in the placebo gp had been intubated or had died: HR in the TCZ gp as compared with the placebo gp: 0.83 (95% confidence interval [CI], 0.38 to 1.81; *p* = 0.64)NS.	Secondary outcomes: Clinical worsening on ordinal scale: HR = 1.11 (95% CI, 0.59 to 2.10; *p* = 0.73). At 14 days, 18.0): NS -Median time to discontinuation of bl supplemental oxygen: 5.0 days (95% CI, 3.8–7.6) in the TCZ gp and 4.9 days (95% CI, 3.8–7.8) in the placebo gp (*p* = 0.69): NS Tertiary outcomes: The median time to improvement: 6.0 days (95% CI, 5.0–6.0) in the TCZ gp and 5.0 days (95% CI, 4.0–7.0) in the placebo gp: NS - The median duration of supplemental oxygen use after administration of TCZ: 4.0 days in the TCZ gp and 3.9 days in the placebo gp. - Admission to ICU or death: 15.9% in the TCZ gp 15.8% in the placebo gp were either admitted to the ICU or died before ICU admission: NS -Median duration of MV: 15.0 days in the TCZ gp and 27.9 days in the placebo gp: NS -Median time to discharge: 6.0 days in both groups: NS	-Neutropenia: 22 in the TCZ gp vs 1 in the placebo gp: *p* = 0.002 -Serious infections: 8.1% in TCZ gp vs. 17.3% in the placebo gp: *p* = 0.03 -36 serious AE in the TCZ gp: 25 unrelated and 11 related or possibly related to TCZ. -38 serious AE in the placebo gp: 35 unrelated and 3 related or possibly related to placebo	51
	-161 in the TCZ						
	-82 in the control gp						
	Age, y-no (%)						
	-60 Gupta et al. (2021a) in the TCZ gp						
	-22 (27) in the TCZ gp						
	-82 Luke et al. (2010) in all patients						
Moderate COVID-19 pneumonia	Total patients 126	1st IV: 8 mg/kg 2^d^ IV: after 12 h - within 8 h from randomization	Alone or with SC of each centre	Clinical worsening within 14 days since randomization: 28.3% in the TCZ arm and 27.0% in the SC gp (rate ratio, 1.05; 95% CI, 0.59-1.86; *p* = 0.87): NS Admission to ICU with MV: 28.3% in the TCZ gp and 27.0% in the SC group: NS Death: 2 patients in the TCZ group vs 1 in the SC gp: NS	-Mortality: At 14 days: 1.7 vs 1.6%; rate ratio, 1.05; 95% CI, 0.07-16.4 -At 30 days: 3.3 vs 1.6%; rate ratio, 2.10; 95% CI, 0.20-22.6 in the TCZ gp vs the SC gp respectively: NS	-23.3% of the AE in the TCZ vs 11.1% in the SC gp. -Increased ALT level and decreased NC in the TCZ gp	53
	-60 in the TCZ gp						
	-66 to the SC gp						
	Age, median (IQR), yr						
	-61.5 (51.5-73.5) in the TCZ gp						
	-60.0 (54.0-69.0) in the SC gp						
	-60.0 (53.0-72.0) in all patients						
Moderate to severe COVID-19 pneumonia	Total patients 131	-Single IV: 8 mg/kg - On day 1 and on day 3 if clinically indicated	SC: Antibiotic agents, AV, corticosteroids. vasopressor support and anticoagulants	-Scores higher than 5 on WHO-CPS on day 4: 12 patients had a WHO-CPS score greater than 5 at day 4 in the TCZ gp vs 19 in the SC gp: (median posterior absolute risk difference [ARD] −9.0%; 90% credible interval [CrI], −21.0 to 3.1): NS -Survival without need of MV at day 14: 24% in the TCZ gp vs 36% in the SC: median posterior HR: [HR] 0.58; 90% CrI, 0.33-1.00: NS	-The evolution of WHO scores during 14-days follow-up: Among patients who were not in ICU at randomization, 18% in the TCZ gp and 22 of 36% in the SC gp were subsequently admitted to the ICU (risk difference, 18%; 95% CI, 0.4–31%): PS -Overall survival, time to discharge, time to oxygen supply independency, biological factors (C-reactive protein level) and AE: Death at day 28: 7 patients in the TCZ gp and 8 in the SC gp: (adjusted HR, 0.92; 95% CI, 0.33-2.53):PS -Overall death with a median follow-up of 28 days: 7 deaths (all from ARDS) in the TCZ gp and 11 (ARDS, n = 9; multiorgan failure, n = 1; pulmonary embolism, n = 1) in the SC gp: NS. -Biological factors: C-reactive protein level and neutrophil count decrease was rapid in the TCZ gp, and lymphocyte count was increased. No patient in the TCZ gp remained with high C-reactive protein level after day 4: PS	-No difference in the occurrence of serious AE between TCZ and SC treatment: 32% patients in the TCZ gp and 43% in the SC gp (*p* = 0.21): NS	38
	-63 in the TCZ gp						
	- 67 in the SC gp						
	Age, median (IQR), y						
	-64.0 (57.1-74.3) in the TCZ gp						
	-63.3 (57.1-72.3)in the SC gp						
Severe COVID-19	Total patients 438	1st IV: 8 mg/kg 2 days: 8–24 h after	AV, low-dose GCC, convalescent plasma, and supportive care. -Within 10 days after COVID-19 symptoms onset.	-Median value for clinical status on 7-category OS at day 28: 1.0 (95% [CI], 1.0 to 1.0) in the TCZ gp and 2.0 (95% CI, 1.0–4.0) in the placebo gp: between-gp difference, −1.0; 95% CI, −2.5 to 0; *p* = 0.31 by the van Elteren test): NS	-Median value for clinical status on 7 category OS at day 28: 3.0 (95% CI, 2.0–4.0) in the TCZ gp and 4.0 (95% CI, 3.0–5.0) in the placebo gp: between-group difference of −1.0 (95% CI, −2.0 to 0.5): NS -Death at day 28: 19.7% in the TCZ gp and in 19.4% in the placebo gp: weighted difference of 0.3 percentage points (95% CI, −7.6 to 8.2; *p* = 0.94): NS. -The median number of ventilator-free days: 22.0 (95% CI, 18.0–28.0) with TCZ and 16.5 (95% CI, 11.0–26.0) with placebo: difference of 5.5 days (95% CI, −2.8–13.0): S -Median no. of days until hospital discharge or readiness for discharge: 20 days (95% CI, 17–27) in the TCZ gp and 28 days (95% CI, 20 to not evaluable) in the placebo gp (Cox proportional-hazards ratio, 1.35; 95% CI, 1.02–1.79): S -Median no. of days until improvement by ≥ 2 categories on 7-category OS in clinical status: 14 days (95% CI, 12–17) in the TCZ gp and 18 days (95% CI, 15–28) in the placebo gp (Cox proportional hazards ratio, 1.26; 95% CI, 0.97–1.64): NS -Median no. of days in the ICU: 9.8 days in the TCZ gp and 15.5 days in the placebo gp, for a difference of 5.8 days (95% CI, –15.0 to 2.9): S --Median no. of ventilator-free days at day 28: 22.0 (95% CI, 18.0–28.0) with TCZ gp and 16.5 (95% CI, 11.0–26.0) with placebo gp: difference of 5.5 days (95% CI, −2.8–13.0): NS -Incidence of MV among patients not receiving MV at randomization: 27.9% in the TCZ gp vs 36.7 in the placebo gp: Difference or HR: (95% CI): 8.9% (–20.7 to 3.0): NS -Clinical failure among patients not receiving MV at randomization: 29.0% in the TCZ gp vs 42.2% in the placebo gp: Difference or HR (95% CI)0.61 (0.40–0.94): NS	-AE: 77.3% of 295 patients in the TCZ gp and in 81.1% of 143 patients in the placebo gp through day 28. -Serious AE in 34.9 and 38.5%, respectively. -Fatal events: 19.7% in the TCZ gp and in 19.6% in the placebo gp through day 28. - 76 serious infections in 21.0% the TCZ gp and 49 25.9% in the placebo gp by day 28. -Similar percentages of patients in each trial gp had AE and serious AE at the clinical cut-off date	49
	-294 in the TCZ gp						
	-144 in the placebo gp						
	Age, mean, y ±SD						
	-60.9 ± 14.6 in the TCZ gp						
	-60 ± 13.7 in the placebo gp						
Reported TCZ Efficacy: RCTs evidences							
Patients with hypoxia and evidence of systemic inflammation	Total patients 4,416	1st IV: 8 mg/kg 2d IV: 8–24 h after. - NR	SC: Corticosteroids alone or plus TCZ.	-28-days mortality: 31% of patients in the TCZ gp vs 35% of 2094 patients in the SC gp; rate ratio 0·85; 95% CI, 0·76–0·94; *p* = 0·0028: S	-Time to discharge from hospital: 57% in the TCZ gp vs 50% in the SC gp; rate ratio 1·22, 1·12–1·33, *p* < 0·0001: S -Progressing to the prespecified composite of invasive mechanical ventilation or death: 35% in the TCZ gp vs 42% in the SC gp: risk ratio 0·84, 0·77–0·92, *p* < 0·0001: S	Three reports of serious AE probably related to TCZ: one each of otitis externa, Staphylococcus aureus bacteraemia, and lung abscess, all of which resolved with SC treatment	47
	-371 in the TCZ gp						
	-4,045 in the SC gp						
	Age, yr, SD						
	-65.8 ± 15.8 and 65.8 ± 15.4 in the TCZ gp						
	-65.8±in the SC gp						
ICU patients under respiratory and/or cardiac mechanical supports	Total patients 865	-1st IV: 8 mg/kg 2^d^ IV: 8–24 h after. - Within 24 h of ICU admission	Alone or with GCC.	-no. of respiratory and cardiovascular organ support–free days up to day 21: 10 in the TCZ gp, 11 in the sarilumab gp, and 0, in the control gp: Median adjusted cumulative odds ratios = 1.64 (95% credible interval, 1.25–2.14), 1.76 (95% credible interval, 1.17–2.91) respectively for TCZ and Sarilumab compared with control. -Posterior probabilities of superiority to control of more than 99.9% and of 99.5%, respectively: S	-90-days survival: 109 deaths in the pooled intervention gp (99 with TCZ and 10 with sarilumab) and 142 in the control gp: HR for the comparison with the control gp of 1.61 (95% credible interval, 1.25–2.08). -Posterior probability of superiority of more than 99.9%: S -Time to ICU discharge: HR with TCZ: 1.42 (95% credible interval, 1.18–1.70) compared to control: S	-2 secondary bacterial infection. -5 bleeding events, -2 cardiac events -1 deterioration in vision NS compared to the control gp	48
	353 in the TCZ gp						
	- 48 to sarilumab gp						
	-402 in the control gp						
	Age, yr, SD						
	-61.5 ± 12.5 in the TCZ gp						
	-63.4 ± 13.4 in the sarilumab gp						
	-61.1 ± 12.8 in the control gp						
	-61.4 ± 12.7 in all patients						
ICU patients	Total patients 3,924	-IV or SC. - First 2 days of admission to the ICU.	Alone or with GCC.	Decreased risk of death: HR, 0.71; 95% CI, 0.56-0.92: S Estimated 30-days mortality: 27.5% (95% CI, 21.2–33.8%) in the TCZ gp and 37.1% (95% CI, 35.5–38.7%) in the non-TCZ treated gp: Risk difference, 9.6%; 95% CI, 3.1–16.0%: S	NR	-Secondary infection: 32.3 vs 31.1%. -AST or ALT level elevation of more than 250 U/L: 16.6 vs 12.9% -AST or ALT elevation of more than 500 U/L: 8.5 vs 5.6%. Arrhythmias: 14.5 vs 17.2% -Thrombotic complications: 10.6 vs 9.8%. NS, respectively in the TCZ the non TCZ treated gps	59
	-433 in the TCZ.						
	-3,491 in the non-TCZ treated gp						
	Age, median (IQR), y						
	-58 (48–65); in the TCZ gp						
	-63 (52–72)in the non-TCZ treated gp						

TCZ: Tocilizumab; yr: years; IQR: interquartile range; SD: standard deviation; HR: hazard ratio; NS: Non-significant effect of TCZ, treatment; PS: Probably Significant effect of TCZ, treatment; S: Significant positive effect of TCZ, treatment; IV: intravenous; SB: subcutaneous (WHO-CPS): World Health Organization 10-point Clinical Progression Scale; NR: not reported; AV: antiviral; HCQ: hydroxychloroquine; MV: mechanical ventilation; BL: baseline; OS: ordinal scale; gp: Group; AE: adverse events; SC: standard care; ALT: Alanine aminotransferase AST: Aspartate aminotransferase; NC: neutrophil count; GCC: glucocorticoids; SR: study reference.

Nevertheless, in another attempt to determine whether tocilizumab improves outcomes of COVID-19 patients hospitalized with moderate-to-severe pneumonia, a multicentre, open-label, randomized clinical trial (CORIMUNO-TOCI 1) included 130 non-ICU hospitalized COVID-19 patients on low flow oxygen support, and excluded patients requiring high-flow oxygen therapy invasive or non-invasive mechanical ventilation, reported that tocilizumab treatment lowered the risk for both invasive and non-invasive mechanical ventilation or mortality by day 14.

However, tocilizumab showed no reduction in progression of the disease at day 4 neither the 28-days mortality rate (Suresh et al., 2021) (Table 1). The median time from symptoms onset to tocilizumab use was 10 (Zhou et al., 2020b; Hoffmann et al., 2020; Huang and Jordan, 2020; Matricardi et al., 2020; Sterne et al., 2020; Tsai et al., 2020; Izda et al., 2021) days in that trial.

An RCT was conducted in 24 hospitals across Italy to assess the efficacy of tocilizumab administration compared to standard therapy in the early course of the infection among COVID-19 hospitalized patients with moderate severity; without the need for invasive or non-invasive mechanical ventilation (Salvarani et al., 2021). A provisional evaluation prematurely ended the study after demonstrating no benefits in reducing mortality and/or clinical progression (Table 1) (Salvarani et al., 2021).

#### 4.2.2 Late Administration in Severely Ill COVID-19 Patients

##### Severe Pneumonia

In moderately ill COVID-19 patients, the COVACTA phase 3 randomized international double-blind placebo-controlled trial (Salama et al., 2021) randomized 452 severe COVID-19 hospitalized patients (blood oxygen saturation ≤93%) to measure the effectiveness and safety of tocilizumab in hospitalized patients with severe COVID-19 pneumonia, but failed to find any significant difference in the clinical outcomes between the tocilizumab treated group and the placebo at day 28. In fact, the study reports no mortality benefits associated with the treatment, in addition to no safety signals with the tocilizumab treatment arm.

Despite the negative results regarding tocilizumab efficacy, percentages of discharged patients, or those ready for discharge, were higher in the treated group compared to the placebo group by day 28, based on the baseline the WHO ordinal scale (Table 1). It is worth noting that patients started the tocilizumab at a frame time of 1–5 days, which was the range of the time from the onset of the symptoms to baseline treatment. However, the study reported no differences in the responses patterns in patients treated earlier rather than later in the course of illness, while other studies did report different responses (Salama et al., 2021).

The randomized, double-blind, phase III placebo-controlled EMPACTA trial that included 389 patients with severe COVID-19 pneumonia requiring only low-flow oxygen therapy showed a positive effect of tocilizumab treatment by reducing the likelihood of progression to mechanical ventilation or death by day 28 (HR 0.56 [95% CI: 0.33-0.97]); however, tocilizumab did not improve survival (EMPACTA, 2020)**.** Of note, more than 50% of the patients in the EMPACTA trial received glucocorticoids compared to the COVACTA and CORIMUNO-TOCI 1 trials.

Table 1 summarises the latest clinical trials and some observational studies findings that studied the use of tocilizumab as an immunomodulator in patients with COVID-19 together with the dose and time of the treatment.

The RECOVERY trial was launched to assess several possible treatments in COVID-19 hospitalized patients in the United Kingdom and specifically targeted those with hypoxia, defined as oxygen saturation <92% on air or requiring oxygen therapy and presenting marks of systemic inflammation such as elevated C-reactive protein levels (≥75 mg/L). Four thousand one hundred and sixteen patients were included in the assessment of tocilizumab. The median time from symptoms onset to tocilizumab treatment was 9 (Zhou et al., 2020b; Hoffmann et al., 2020; Huang and Jordan, 2020; Matricardi et al., 2020; Sterne et al., 2020; Tsai et al., 2020; Izda et al., 2021) days. Overall, tocilizumab was associated with a significant reduction in 28-days mortality (risk ratio 0.85 [95% CI: 0.76-0.94]). Moreover, progression to invasive mechanical ventilation or death was less likely among patients not receiving invasive mechanical and treated by tocilizumab. Of note, 82% of the patients were receiving systemic corticosteroids.

The results of this study were consistent among different prespecified subgroups such as respiratory support, and days since symptoms onset, except in the use of corticosteroids subgroup where the efficacy of tocilizumab was observed only in patients who received systemic corticosteroids (RECOVERY Collaborative GroupHorby et al., 2021).

##### Intensive Care Unit (ICU) Patients

In the context of critically ill COVID-19 patients, ICU patients receiving respiratory or cardiovascular organ support have gained the attention of multiple trials in order to assess the effect of tocilizumab at this stage of advanced disease. The Randomized, Embedded, Multifactorial Adaptive Platform Trial for Community-Acquired Pneumonia (REMAP-CAP) study is an international trial that compared the treatment of COVID-19 in critically ill patients with tocilizumab or sarilumab, to patients receiving standard therapy. Either treatment was administered within 24 h after starting organ support in the ICU.

In this study, patients on respiratory support were categorized as those receiving invasive or non-invasive mechanical ventilation, including through high-flow nasal cannula, if the flow rate was more than 30 L/min and the fraction of inspired oxygen was more than 0.4. Patients on cardiac support were those receicing any vasopressors or inotropes. Eight-hundred-three critically ill patients were included in this study. Tocilizumab was administered on the first day of ICU admission. Results showed that both tested IL-6R antagonists improved days free of organ support up to 21 days and 90-days survival (Table 1). The study has also demonstrated that both immunomodulatory treatments showed better-estimated effects when administered with glucocorticoids than monotherapy of either drug. Moreover, the study demonstrated that the interaction between either tocilizumab or sarilumab and glucocorticoids was additive and slightly in the direction of synergistic (Gordon et al., 2021).

## 5 Discussion

Previous paragraphs of the review discussed high concentrations of IL-6 as an important cytokine in the pathophysiology of the SARS-CoV-2- induced cytokine storm and a prognostic factor in COVID-19 infection (Del Valle et al., 2020b). Nevertheless, it is still contradictory whether fundamental differences in effects exist between IL-6 antagonism and IL-6R antagonism, respectively, using siltuximab to inhibit both cis and trans IL-6 signaling and tocilizumab to block both mIL-6R and sIL-6R and all the subsequent cis and trans singling and trans presentation. However, Trans presentation involves IL-6 binding to mIL-6R expressed on an immune cell to form a complex with gp130 on T helper 17 (TH17) cells, leading to downstream T cell signaling involved in ARDS (Moore and June 2020b). Moreover, it has been well documented that IL-6 inhibitors do not have action on IL-6 produced by all kinds of viruses, which is the case of human herpesvirus-8 and HIV (Arnaldez et al., 2020). Tocilizumab is currently standing out as an IL-6 receptor blockade that might interrupt the inflammatory cascade at a crucial stage. Both early and ongoing research initiatives have demonstrated promising potential and relative validated benefits (Guaraldi et al., 2020b; Gupta et al., 2021b) with more than 25 completed clinical trials (ClinicalTrials, 2021), unlike scarce clinical data about other types of immunomodulators. Moreover, recent guidelines such as the NIH’s, have introduced tocilizumab as a panel recommended immunomodulator. JAK inhibitors have also been proposed as COVID-19 potential treatments based on their ability to prevent the phosphorylation of signal transducer and activator of transcription (STAT) proteins involved in the immune activation and inflammation (Babon et al., 2014), such as cellular response to proinflammatory cytokines like IL-6 (Zhang et al., 2020b). Moreover, JAK inhibitors also have a direct antiviral activity that prevents viral endocytosis, which makes them potentially capable of preventing the SARS-CoV-2 from entering and infecting target cells (Stebbing et al., 2020). Positive panel’s recommendations about Baricitinib and Tofacitinib and their negative recommendations about Ruxolitinib are based on few available clinical data from, mainly, ACTT-2 (Kalil et al., 2021), COV-BARRIER (Marconi et al., 2021), and STOP-COVID (Guimarães et al., 2021) clinical trials. The first trial showed improved time to recovery after treatment with baricitinib given in combination with remdesivir to hospitalized patients with COVID-19 who require supplemental oxygen but not mechanical ventilation. However, the effect of baricitinib when given in addition to corticosteroids has not been evaluated.

A second example is systemic corticosteroid therapy that has been shown to improve clinical outcomes and reduces mortality in COVID-19 hospitalized patients requiring supplemental oxygen therapy (Sterne et al., 2020). Corticosteroid therapy is presumably capable of lessening the severity of the SARS-CoV-2-induced systemic inflammatory response, thus preventing lung injury and multiorgan dysfunction with no observed benefit in hospitalized patients who do not require supplemental oxygen therapy (RECOVERY Collaborative GroupHorby et al., 2021). Positive COVID-19 Treatment Guidelines Panel’s recommendations for using corticosteroids in hospitalized patients with COVID-19 stem from 7 completed clinical trials. However, no data support their use in COVID-19 non hospitalized patients.

On the other hand, tocilizumab has been largely approved by the FDA and highly recommended by the panel for the treatement of COVID-19 in hospitalized patients (Characteristics of Immunomodulators, 2022). Different meta-analyses and systematic reviews were published on the efficacy of tocilizumab treatment in COVID-19 patients with contradictory findings (Ghosn et al., 2021; Shankar-Hari et al., 2021; Snow et al., 2021). The latest and largest was the Rapid Evidence Appraisal for COVID-19 Therapies (REACT) meta-analysis that included 27 RCTs with a total of 10 930 COVID-19 patients and 6,449 patients treated with IL-6r antagonists (IL-6Ra), representing 90% of all COVID-19 patients registered in IL-6Ra research trials (Shankar-Hari et al., 2021). The meta-analysis reported a significant reduction in all-cause 28-days mortality compared to placebo or standard care for tocilizumab (OR: 0.83 [95% CI:0.74-0.92]). Moreover, most of the reported RCTs were able to distinguish groups of patients receiving corticosteroids at randomization (22 trials). Interestingly, there was a significant interaction between IL-6 antagonists and corticosteroids. Indeed, concomitant administration of corticosteroids and tocilizumab resulted in further decreases in the 28-days all-cause mortality compared to placebo or standard of care (OR: 0.77 [95% CI: 0.68-0.87]). Also, concomitant use of tocilizumab and corticosteroids resulted in less likelihood of progression to invasive mechanical ventilation, ECMO, or death at 28-days compared to placebo or standard of care (OR: 0.69 [95% CI: 0.61-0.78]). However, tocilizumab alone with no corticosteroid use did not significantly improve mortality or decrease the likelihood of clinical worsening compared to placebo or standard of care.

Based on REACT findings, tocilizumab resulted in lower 28-days mortality in patients requiring oxygen ≤15 ml/min (OR: 0.82 [95% CI:0.67-1.00]) and non-invasive ventilation (OR: 0.80 [95% CI: 0.68-0.93]), but not in patients receiving invasive mechanical ventilation at randomization (OR: 0.92 [95% CI: 0.72-1.17]), consistent with the inverse association of progression to invasive mechanical ventilation or death among these patients. However, the interaction between tocilizumab and respiratory support was not statistically significant (*p* = 0.43), suggesting that these differences between the respiratory support subgroups might be due to sampling variation.

Also, tocilizumab did not show any beneficial effects on reducing the 90-days mortality rate or duration of invasive mechanical ventilation among patients receiving invasive mechanical ventilation at randomization, but data sets were small to draw these conclusions. Moreover, tocilizumab efficacy was not affected by the need for vasopressors, age, sex, race, or patients’ ethnicity (Shankar-Hari et al., 2021).

Interestingly, the beneficial effects on outcomes associated with tocilizumab did not differ according to the level of C-reactive protein in this prospective meta-analysis, which is consistent with the REMAP-CAP trial findings (Gordon et al., 2021; Shankar-Hari et al., 2021). There might be two possible explanations for this finding; First, systemic inflammation may not be a good discriminant indicator for the tocilizumab effects, and local inflammation can be, as evidenced by acute respiratory failure, more useful as a marker of which patients would benefit.

In this regard, systemic cytokines levels in COVID-19 were lower than with other causes of sepsis and acute respiratory distress syndrome (Leisman et al., 2020). Second, the C-reactive protein may not be the perfect biomarker for such stratification.

The optimal timing of tocilizumab use was not addressed in the prospective REACT meta-analysis (Shankar-Hari et al., 2021). However, the REMAP-CAP trial demonstrated a hazard ratio of 1.59 (95% C: 1.24-2.05) for increased 90-days probability survival in patients who received tocilizumab in the first 24 h after ICU admission (Gordon et al., 2021). This suggests that tocilizumab is most effective when administered early in the disease course, while any developing organ dysfunction may be more reversible. Optimal timing of tocilizumab adminstartion is still controversial, it requires further verification by conducting timing-related RCTs.

One of the most critical limitations of the REACT meta-analysis is the absence of accounting for the baseline severity of respiratory failure or organ dysfunction and, therefore, the baseline risk of death. Indeed, the benefits of tocilizumab may not translate to patients with a low baseline mortality risk, modest oxygen requirements, and a stable clinical course. Nevertheless, despite this conflicting evidence, the Panel’s recommendations for using tocilizumab are based on the collective evidence from the clinical trials reported to date.

In summary, and in accordance with the NIH guidelines for therapeutic management of hospitalized adults with COVID-19:Therapeutic Management of Hospitalazied Adults With COVID-19 Treatment Guidelines (nih.gov), tocilizumab should be given early (within 24 h of organ failure) in patients with substantial oxygen requirements and progressive disease while receiving glucocorticoids irrespectively of C-reactive protein levels.

While presenting promising benefits, tocilizumab administration should be balanced against potential associated adverse events, including hepatic toxicity, thrombocytopenia, thrombosis, bleeding, and many others.

Although some retrospective studies concluded that tocilizumab was associated with new other infections, RCTs showed that despite the higher incidence of leucopenia among patients treated with tocilizumab (13.7 vs. 1.2%, *p* = 0.002), serious infections occurred in fewer patients compared with placebo (9.1 vs. 17.1%, *p* = 0.03) (Xu et al., 2020; Salama et al., 2021). Also, in almost all the RCTs, adverse events were similar between tocilizumab and non-tocilizumab groups, with some of them reporting fewer secondary infections. Furthermore, the REACT meta-analysis reported no significant increase in the risk of secondary infections at 28 days among the vast number of patients included, suggesting that the use of tocilizumab is safe in these patients (Shankar-Hari et al., 2021). However, this finding should be interpreted with caution, given that the number of reported events is lower than it might be expected.

## 6 Conclusion

Tocilizumab is a promising treatment for COVID-19 hospitalized patients with progressive disease and high oxygen requirements. However, it is not yet warranted for extensive use in patients with mild disease or prolonged invasive mechanical ventilation.

## Abbreviations

ADAM17, Disintegrin and metalloprotease 17; IL-6, Interleukin-6; IL-6R, IL-6 receptor; IL-6Ra, IL-6r antagonists; ACE2, Angiotensin-converting enzyme 2; ARDS, Acute respiratory distress syndrome; GM-CSF, Granulocyte-macrophage colony-stimulating factor; TNF-α, Tumor necrosis factor-α; CRS, Cytokine release syndrome; AngII-AT1R, Angiotensin II-angiotensin receptor type 1; sIL-6Ra, Soluble form of interleukin-6 receptor alpha; sHLH, Secondary hemophagocytic lymphohistiocytosis; IP-10, Interferon gamma-induced protein 10; MIP1A, Macrophage inflammatory protein 1 alpha; GM-CSF, Granulocyte-macrophage colony-stimulating factor; JAKs, Janus kinases; NK, Natural killer; RCTs, Randomized control trials; ICU, Intensive Care Unit; STAT3, Signal transducer and activator of transcription 3.
